# The Necessity of an Organized Inspection of Our Meat and Milk Supply by Educated Veterinarians

**Published:** 1885-10

**Authors:** 


### EDITORIAL DEPARTMENT.

### THE NECESSITY OF AN ORGANIZED INSPECTION OF OUR MEAT AND MILK SUPPLY, BY EDUCATED VETERINARIANS.

Although we have, in almost every issue of this journal, referred to these important questions, and, in our last, published a sketch of the methods in vogue at the best conducted abattoir in the world, we do not seem to have been successful in planting any fruitful seed. One of the most surprising phenomena in this connection is, that none of the medical journals in this country have either noticed these articles or given that attention to the subject which they should do as guardians of the public health.

It has thus far been impossible, both by personal advocacy and reiterated appeals in writing, to make any impression upon Boards of Health.

Neither the State or city representatives of the people seem able to realize a fact that has long been patent to the intelligence of the same class of men in Europe, *i. e.*, that none but qualified veterinarians can be trusted to do this duty properly.

Such authorities assume that any politician is much better suited to guard the public health, in these important matters, than men whose professional studies especially adapt them for these responsibilities. They do not seem to be aware that the educated veterinarian knows that the advancement of his profession depends upon his being strictly honest in such duties.

Our attention has been again called to these questions, by reading the following remarks in a number of Chicago daily papers :

> The article says : "The health inspectors are apparently in ignorance of the fact that diseased sheep are almost daily sold at the stock-yards. Sales are made openly, despite the presence of the inspectors. and no bar has thus far been interposed to stop the repulsive traffic. The animals are shocking spectacles, and the idea that such stuff is sold daily as food for thousands is horrifying. A thousand diseased sheep are bought at the stock-yards daily by mercenary 'scalpers,' and sold to the unsuspecting public as good mutton. A syndicate is interested in the traffic."

Now, who consumes such meat? The poor and needy! who, if they cannot have luxuries, should have what they use as food guaranteed to them to be of the healthiest quality.

As a supplement to the above we may say, that we know of butchers who sell entirely among this class of our citizens, that make it a practice to buy nothing else but diseased animals, the flesh of which they peddle about from their carts, in the face of the authorities. Such animals never find their way to the inspectors, but are slaughtered in sheds, barns or out-of-the-way places on the confines of our cities.

Hence the need of a public abattoir in every city. Hence the need of the most stringent laws forbidding slaughtering anywhere else.

We pretend to have an inspection of the milk supply of our cities; yet, in spite of it, the veterinarians of any of them could easily show the Boards of Health where hundreds of illy-fed, poorly-housed cattle are packed together in hovels, with filth above their ankles, breathing a stench almost impossible to endure, and almost every cow the subject of tuberculosis.

We recently went the rounds, with the meat inspector, of a neighboring city, and came across a poor, emaciated cow—"a new milch cow"—with a calf by her side; the cow was pretty well gone with tuberculosis, and what made her especially interesting was, she also had manifest tuberculosis of the udder.

We asked the inspector "what he was going to do under the circumstances?" His answer was: "I can do nothing; the law does not recognize these cases." The cow was sold; went into a poor neighborhood, and her milk was peddled out to deal the seeds of that fellest of all destroyers among the children in the vicinity, consumption.

The Government of the United States says, "American hogs are almost free from trichinæ," yet one can read nearly every week of persons dying from the effects of this parasite.

Mr. Redding, whose Report to the Indiana Board of Health we have reviewed in this issue, says: "The highest percentage of infested hogs were those of the retail butchers."

We have examined more American pork than all the other examiners together, and found four per cent. trichinous.

